# Active screen time and imagination in 5–6-years-old children

**DOI:** 10.3389/fpsyg.2023.1197540

**Published:** 2023-05-15

**Authors:** Daria Bukhalenkova, Olga Almazova

**Affiliations:** ^1^Department of Educational Psychology and Pedagogy, Faculty of Psychology, Lomonosov Moscow State University, Moscow, Russia; ^2^Department of Developmental Psychology, Faculty of Psychology, Lomonosov Moscow State University, Moscow, Russia

**Keywords:** preschool age, imagination, creativity, flexibility, originality, elaboration

## Abstract

This research focused on the connection between such factors of the active screen time of preschoolers as the time spent playing computer games and parental participation in children’s computer games on imagination in 5–6 years old children. The mothers of 772 children were asked to fill out questionnaires where they described how their children interact with gadgets. 371 of these children also participated in the test that assessed productive imagination using complete the drawing task (such parameters as flexibility, originality, elaboration were assessed). As a result of the study, no relationship was found between imagination and the time spent by preschoolers playing computer games. At the same time, this study revealed significant relationships between imagination and the characteristics of parental participation in the gadgets’ usage by preschoolers. The research showed that imagination flexibility scores are significantly higher in children who use gadgets with siblings or peers than in those who often play alone or with an adult.

## 1. Introduction

One of the most important aspects of cognitive development in preschool age is imagination, which is significant for the children’s further successful development and learning (Vygotsky, 1984; Lubart, 1999; Alfonso-Benlliure et al., 2013; Calvert, 2015; Gajda et al., 2016; Chen et al., 2020; Bayanova and Khamatvaleeva, 2022). At preschool age, imagination actively develops within the role play (Vygotsky, 1984), however in the modern world, traditional play with peers among preschoolers is supplanted and supplemented by the active use of gadgets (Singer and Singer, 2005; Calvert, 2015; Götz, 2015; Kalabina and Progackaya, 2021; Belova and Shumakova, 2022; Yudina, 2022). At the same time, some scientists adhere to the hypothesis that gadgets have a developing potential for imagination (for example, Jackson et al., 2011; Ott and Pozzi, 2012; Götz, 2015; Blanco-Herrera et al., 2019); whereas others support the opposite idea that gadgets are more likely to impoverish the imagination (e.g., Singer and Singer, 2005; Greenfield, 2009; Calvert and Valkenburg, 2013). In this regard, studying the gadgets’ influence on the imagination development in preschoolers remains relevant and significant.

It is the active screen time (i.e., playing on smart electronic devices) and not the passive screen time (i.e., watching cartoons and various video content on television/tablet/mobile phone), that seems to be the most significant in terms of the cognitive development of preschoolers (Linebarger et al., 2014; McNeill et al., 2019; Veraksa N. E. et al., 2021) and of the imagination too (Calvert, 2015). In a computer game, children have more opportunities to show initiative, activity and independence than when watching a cartoon with a predetermined plot. Such active participation in a computer game, on the one hand, trains various cognitive functions that all are interconnected with the imagination (since all mental functions develop in a systemic and interconnected manner) (Vygotsky, 1984). On the other hand, the need to choose a strategy, think over your actions and their possible consequences, find ways to solve problems in computer games stimulate children creativity and imagination. Numerous studies convincingly show the potential of popular children’s computer games and specially designed computer programs (serious games) to develop creativity and imagination in children (Cassell and Ryokai, 2001; Bertolini and Nissim, 2002; Kannetis et al., 2009; Jackson et al., 2011; Ott and Pozzi, 2012; Blanco-Herrera et al., 2019; Papadakis, 2020; Rahimi and Shute, 2021; Xiong et al., 2022).

However, such educational computer programs and applications are not always available to parents because they are developed as part of research and are not always in the public domain. In addition, parents do not always know how to choose educational applications for their children and optimize play time (Broekman et al., 2016; Brito and Dias, 2020; Veraksa A. N. et al., 2021; Khokhlova et al., 2022). Thus, it is essential to study the impact that ordinary, everyday (and not created within the study) gadgets’ usage by children has on their creativity. Based on all the reasons described, this research focused on the influence of such factors as the game’s duration and parental participation in children’s computer games on imagination in 5–6 years old children.

The results of studies of the relationship between time spent on computer games and creativity or imagination in preschoolers are quite contradictory. Some studies show no relationship between time spent on games and creativity (Hamlen, 2013), while others show a positive relationship between the two (Jackson et al., 2011). Data on the gadgets’ negative impact on creativity was obtained mainly about the time spent watching TV, and not the time spent on computer games by children (Valkenburg and van der Voort, 1995; Calvert, 2015). Based on the previously obtained data on the relationship between gadgets’ use and other cognitive functions (Bowers and Berland, 2013; Soldatova and Vishneva, 2019), it can be assumed that such a contradiction in the results may be due to the non-linearity of this relationship. It is likely that there is some optimal amount of time to spend playing computer games that increases the level of imagination, while the complete lack of playtime with gadgets or excessive playing time will reduce creativity scores in preschoolers.

As for the research on parent mediation in the gadgets’ usage by children, these studies normally focus either on parental beliefs about the benefits or harms that can bring the usage of different computer games and applications by children, or they focus on parental educational strategies regarding the rules for using gadgets by children (Broekman et al., 2016; Palaigeorgiou and Katerina, 2017; Brito and Dias, 2020). Quite a lot of research has been devoted to this issue, and based on those specific recommendations have been formulated for parents regarding the digital devices’ use by children (for example, Blum-Ross and Livingstone, 2017). At the same time, the role of the joint play of a child with an adult with gadgets at preschool age is practically not researched at all.

It is crucial to mention that one of the problems in research on creativity is that the authors define this concept and the phenomena it describes differently, and therefore use different methods to assess it (Sternberg and Lubart, 1999; Runco and Jaeger, 2012; Williams et al., 2016). This leads to additional complexity when comparing and interpreting the results of different studies on this topic. In this study, we rely on the most widespread understanding of creative imagination (i.e., creativity) in child psychology as a special ability of a person to create something objectively and/or subjectively new and at the same time corresponding to the requirements of the situation (Dyachenko, 1996; Sternberg and Lubart, 1999; Calvert, 2015).

Thus, data on the influence of such factors as the time spent playing computer games and parental participation in children’s computer games on imagination are rather small and contradictory. In addition, a large number of studies on this topic are devoted to older children - schoolchildren and adolescents, and not to preschoolers (Jackson et al., 2011; Hamlen, 2013). To complement the scientific data available on this topic, the purpose of this research was to trace whether the imagination level differs in children interacting with digital devices for a different amount of time and how adults’ participation in children’s interactions with gadgets is correlated with their level of imagination.

## 2. Materials and methods

### 2.1. Sample

The mothers of 772 children were asked to fill out questionnaires where they described how their children interact with gadgets, among the study participants there were 390 (50.5%) boys and 382 (49.5%) girls aged 58–73 months (*M* = 65.3; SD = 3.99).

Of the half of those for whom the parents answered (386 children), a subsample was drawn up, in which the distribution by sex and age of children coincided with the general sample. With them, a technique to assess the imagination was carried out. The results of 15 children were excluded from the analysis due to the child’s refusal to complete the task or misunderstanding of the instructions. As a result, the sample of tested children was 371, among them were 175 (47.2%) boys and 196 (52.8%) girls aged 59–71 months (*M* = 65.2; SD = 3.84).

### 2.2. Measures

To study the peculiarities of the gadgets’ usage by preschoolers, a questionnaire for parents was used. The questionnaire consisted of several blocks of questions about SES, peculiarities of the family situation, peculiarities of children’s use of gadgets, and peculiarities of children’s behavior. In this research, 3 questions from the questionnaire about the playing computer games were analyzed. In the first two questions, parents were asked to indicate the number of hours and minutes that a child usually spends on electronic devices on weekdays and weekends separately, excluding the time spent watching cartoons and videos («How much time on a typical weekday/weekends does a child spend on electronic devices (computer, tablet, phone, game console), not counting the time spent watching cartoons and videos?»). The third question was “Who more often decides what games a child will play on an electronic device?” and the following 3 answers were offered to parents: (1) “More often I or other adult family members”; (2) “More often the child”; and (3) “The child does not play games on the electronic device.” In the fourth question, parents were asked “With whom does the child usually spend time playing electronic devices?” - parents were offered the following answers: (1) “alone”; (2) “with brothers/sisters”; (3) “with adult family members”; (4) “the child does not play games on the electronic device”; (5) “other (please, specify).” Parents of preschool children completed the questionnaire individually.

To assess the preschoolers’ imagination, the “Complete the Drawing” test was used, which is a modified version of the test by Torrance (1962) and Dyachenko (1996). It is widely used in Russia and is the most common test amongst the researchers in the field of preschool development to measure the children’s imagination level.

**FIGURE 1:**
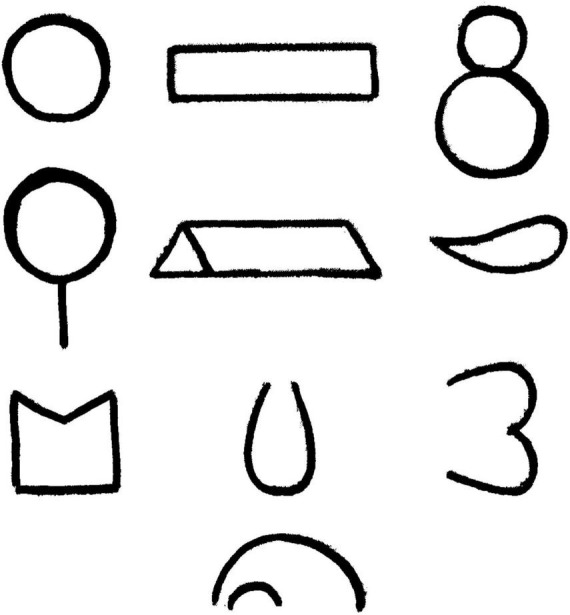
Tasks of the technique “complete the drawing” (10 figures; Dyachenko, 1996). Reproduced with permission.

The test included 10 cards, and each card had one figure of indefinite shape drawn on it (see Figure 1). The task of a test participant was to finish each drawn figure so that a completed image was created. The test results were evaluated according to four indicators:

(1) Images’ originality–the number of the original figure inclusions in the new graphic image. An image is considered original if the initially given figure plays the role of an insignificant component (for example, a triangle is not a roof of a house, but a pencil lead with which a boy draws a picture, etc.). The total number of drawings with inclusions was calculated (maximum 10 points).
(2) Images’ elaboration–the level of detail, i.e., the number of elements added by the child. This indicator reflects the child’s ability to develop his/her ideas in detail. This indicator was calculated separately for each image, then the average score was calculated for all the drawings of each participant.
(3) Imagination flexibility–the number of non-repeating (in terms of content and drawing principle) images for each child. Images were considered identical, in which the initially given for the drawing figure turned into the same element (for example, a child draws the figures “circle” and “circle with a stick” like a lollipop). Thus, for this parameter, each participant could score a maximum of 10 points.
(4) Originality coefficient–the number of unique images that are different from other drawings of the same child, as well as from the drawings of other children from his/her kindergarten group, drawn on the basis of the same initially given figure (maximum 10 points).

### 2.3. Procedure

Each child was tested individually in a quiet and bright room of the kindergarten where he/she was studying. Tests were facilitated by a specially trained tester. Children were free to quit or refuse to participate in the research at any time as well as were explicitly asked about their desire to participate in the research.

All parents were informed about the research objectives and gave written consent for children’s involvement in the study. The research was approved by the Ethics Committee of the Faculty of Psychology at Lomonosov Moscow State University (the approval No: 2022/15).

## 3. Results

### 3.1. Gadgets’ use of preschoolers

Based on the data obtained, we calculated how many minutes approximately per week a child actively uses gadgets: from 0 to 1920 min (*M* = 555.8; SD = 681.47). Since we assumed a non-linear relationship between the time spent playing with gadgets and imagination, for further analysis, the children were divided into 3 groups, approximately equal in size, based on the frequency table: rare, medium and frequent use of gadgets (see Table 1). The number of minutes in all three selected groups differs significantly (Anova, *F* = 388.393, *p* < 0.001).

**TABLE 1:** Selected groups of children with different frequency of active gadgets use.

Frequency	Range (min)	% children
Rare	Up to 210	31.1
Medium	From 210 to 570	34.6
Frequent	More than 570	34.2

An analysis of the answers to the question about who decides more often what games a child will play on an electronic device showed that children themselves more often determine the games they will play (56.0% of answers), rather than parents (24.7% of answers). Mothers were also asked with whom a child usually watches videos and plays with. According to the data obtained, the majority of children play with a sibling or friend (37.3%) or alone (31.7%), and only 12.0% of children play with adults. At the same time, about 19.3% of children do not play with gadgets at all according to the answers given by their mothers.

### 3.2. Imagination of preschoolers

The results of the imagination indicators assessing of preschoolers (elaboration, originality, coefficient of originality and imagination flexibility) showed that in terms of imagination flexibility, we observed a small data scatter, showing that most children demonstrate high imagination flexibility level: the majority of participants made 9 or all (10) non-repeating (in content and drawing principle) images, when they were presented with 10 unfinished drawings within the test (see Table 2).

**TABLE 2:** Means, medians and standard deviations of scores according to the “complete the drawing” method.

	*M*	Me	SD	Min.	Max.
Elaboration	7.66	7.00	3.170	2	21
Originality	1.25	1.00	1.205	0	6
Originality coefficient	4.40	4.00	1.756	0	9
Flexibility	9.63	10.00	0.694	6	10

The Kolmogorov–Smirnov test showed that the distribution according to the imagination’s different aspects was not normal, which indicates the need for further analysis with non-parametric criteria.

### 3.3. Preschoolers’ imagination in connection with the gadgets’ usage peculiarities

Firstly, we compared the imagination assessment scores in different groups of children based on the gadgets’ usage peculiarities highlighted in the analysis (using the Kruskal–Wallis test for several independent samples). There were no significant differences in the imagination parameters depending on the gadget frequency use (rare, medium, frequent use).

Secondly, we have found that imagination elaboration scores were significantly higher in children who chose content themselves more often than in children whose content was chosen by adults (Mann–Whitney test, *U* = 7633.5, *p* = 0.035) (see Table 3). Also, imagination flexibility scores were significantly higher in children who used gadgets together with siblings or peers than in those who played alone (*U* = 7510.0; *p* = 0.046) or with an adult (*U* = 2457.5; *p* = 0.014) more often (see Table 4).

**TABLE 3:** Differences in imagination parameters depending on who chooses the content (child or adult).

	Who chooses the content				Differences	
	Child		Adult			
	*M*	SD	*M*	SD	*U*	*P*
Elaboration	7.84	3.432	6.96	2.766	7633.5	0.035
Originality	1.29	1.273	1.04	1.017	8374.5	0.217
Originality coefficient	4.32	1.738	4.39	1.820	8977.5	0.762
Flexibility	9.63	9.67	9.67	0.565	9060.0	0.819

**TABLE 4:** Differences in imagination parameters depending on with whom the child uses gadgets.

	Alone		With peers or siblings		With parents		Differences	
	***M***	**SD**	***M***	**SD**	***M***	**SD**	**K-W**	***p***
Elaboration	7.74	3.351	7.72	3.289	7.00	2.88	0.872	0.647
Originality	1.17	1.162	1.30	1.224	1.21	1.353	0.955	0.620
Originality coefficient	4.14	1.771	4.47	1.793	4.60	1.781	3.456	0.178
Flexibility	9.57	0.789	9.75	0.535	9.43	0.914	7.292	0.026

## 4. Discussion

The purpose of this study was to see if the imagination differed in with different amounts of active screen time, and how the participation of adults and peers in the interactions of children with gadgets connected with their imagination.

As a result no correlation was found between the time a child spends playing computer games and imagination indicators, which is consistent with the data of the Hamlen (2013) study, however, this data was obtained on a sample of 12-year-old children. The data generated within our study do not support the assumption that there is some optimal amount of time playing computer games that increases the imagination level, while the complete lack of play time with gadgets or excessive play time will reduce imagination level in preschoolers. This result suggests that from the imagination development in preschoolers point of view, not just the time that a child spends playing computer games is important, rather the content of child’s activity via gadgets (Veraksa et al., 2022).

At the same time, our study revealed significant relationships between imagination and the characteristics of parental participation in the gadgets’ usage by preschoolers. It was discovered that the drawings’ elaboration level was significantly higher in children who chose the content to play with via gadgets themselves more often than in children whose content was chosen by adults. This result suggests that children who have more freedom in choosing computer games are better able to develop their ideas in detail. This finding is consistent with the theory that video game-induced positive emotions contribute to imagination in preschool age (Hutton and Sundar, 2010). This result also shows that parents are not always competent in choosing children’s games in terms of their importance for the preschoolers’ imagination development (Broekman et al., 2016). Probably a child’s enthusiasm and interest in play is more essential in this context (Kannetis et al., 2009). If the images of computer characters are attractive, children can actively include them in their fantasies, and thus computer games can contribute to the imagination development (Götz, 2015).

According to the research results, imagination flexibility scores are significantly higher in children who use gadgets together with siblings or peers than in those who often play alone or with an adult. This result is of particular interest. It can be assumed that in a joint game with a child, an adult takes the position of an observer of the child’s play, rather than acting as an equal participant in the game. At the same time, when playing with a peer or sibling, the child often has to agree with him/her on the rules for playing together or using the gadget, which trains the preschoolers’ executive functions that closely related to the development of imagination and creativity (Krumm et al., 2018; Filippetti and Krumm, 2020; Veraksa et al., 2020). On the other hand, according to Vygotsky (1984), children often act out in a role-playing game the life experiences they have received and vivid impressions from the events that have happened to them. Then the impressions and emotions received in a computer game can become the basis for a joint game with peers and fantasizing, contributing to the development of children imagination (Fleer, 2022). It can also be assumed that a more active participation of adults in a children’s computer games (discussing with children what is happening in a computer game, suggesting ways to solve tasks, etc.) would have a more developing effect on children imagination (Vygotsky, 1984; Strouse et al., 2013).

Speaking about the limitations of the study, it is important to note that this study did not analyze other aspects of gadgets use [for example, the role of passive screen time (Greenfield et al., 1986; McNeill et al., 2019)]; additional variables related to imagination were not taken into account [for example, personality traits and children’s cognitive abilities, social and educational factors (Sternberg and Lubart, 1999; Lucchiari et al., 2019; Yildiz and Yildiz, 2021; Tvardovskaya et al., 2022)]. Also, the limitations of this study include the research methods’ specifics that we have chosen. Firstly, via the “Complete the Drawing” test it is not possible to evaluate some types of activity of preschoolers, in which their imagination can also manifest itself [for example, the children’s ability to make up stories (Dyachenko, 1996; Sternberg and Lubart, 1999)]. Secondly, in this study we measured the time that preschoolers spent using gadgets based on a parents’ survey, and not on the observation diaries filled in by parents, which is a more reliable and secure way to measure this parameter (Calvert, 2015). However, this method requires a lot of time to be spent by parents, which reduces the likelihood of parents participating in the study and, accordingly, collecting a large amount of data. This specific approach toward the time estimation, as well as possible differences in parents’ perceptions of what exactly a child is doing with the help of a gadget (playing or studying) (Calvert, 2015) can significantly affect the research results, which indicates the need for further studying of this topic and findings verification. Furthermore, a survey of parents did not allow us to find out the characteristics of the games that children play (age-appropriate or not, game type, educational component), which is also of great importance for the imagination development in preschool age (Calvert, 2015; Papadakis, 2020; Xiong et al., 2022). In the future, we plan to analyze the computer games’ type and content based on interviews with children.

Therefore, the research expands the available scientific knowledge about the relationship between the gadgets’ usage specifics and imagination in modern preschoolers. Based on the results obtained, it can be concluded that the amount of time that a preschooler spends playing computer games itself is not important, rather with whom and what he/she plays.

## Data availability statement

The raw data supporting the conclusions of this article will be made available by the authors, without undue reservation.

## Ethics statement

The studies involving human participants were reviewed and approved by the Ethics Committee of the Faculty of Psychology at Lomonosov Moscow State University (the approval No: 2022/15). Written informed consent to participate in this study was provided by the participants’ legal guardian/next of kin.

## Author contributions

DB was involved in the data collection. OA verified the analytical methods and conducted the analyses. DB wrote the manuscript with critical feedback and input from OA. Both authors discussed the results and contributed to the presented idea (i.e., research questions) and the theoretical framework, and approved the submitted version.

## References

[B1] Alfonso-Benlliure V. Mélendez J. C. García-Ballesteros M. (2013). Evaluation of a creativity intervention program for preschoolers. *Think. Skills Creativ.* 10 112–120. 10.1016/j.tsc.2013.07.005

[B2] Bayanova L. F. Khamatvaleeva D. G. (2022). Review of foreign research on creative thinking in developmental psychology. *Moscow Univ. Psychol. Bull.* 2 51–72. 10.11621/vsp.2022.02.03

[B3] Belova E. S. Shumakova N. B. (2022). Features of the use of digital devices as components of a family microenvironment for the cognitive development of older preschoolers. *Presch. Educ. Today* 6 42–53.

[B4] Bertolini R. Nissim S. (2002). Video games and children’s imagination. *J. Child Psychother*. 28, 305–325. 10.1080/0075417021000022667

[B5] Blanco-Herrera J. A. Gentile D. A. Rokkum J. N. (2019). Video games can increase creativity, but with caveats. *Creativ. Res. J.* 31 119–131. 10.1080/10400419.2019.1594524

[B6] Blum-Ross A. Livingstone S. (2017). *Families and screen time: Current advice and emerging research.* London: London School of Economics and Political Science, 52.

[B7] Bowers A. Berland M. (2013). Does recreational computer use affect high school achievement? *Educ. Technol. Res. Dev.* 61 51–69. 10.1007/s11423-012-9274-1

[B8] Brito R. Dias P. (2020). “Which apps are good for my children?”: How the parents of young children select apps. *Int. J. Child-Comp. Interact.* 26:100188. 10.1016/j.ijcci.2020.100188

[B9] Broekman F. L. Piotrowski J. T. Beentjes H. W. J. Valkenburg P. M. A. (2016). Parental perspective on apps for young children. *Comp. Hum. Behav.* 63 142–151. 10.1016/j.chb.2016.05.017

[B10] Calvert S. L. (2015). “Children and digital media. CHAPTER 10,” in *Media, imaginative play, creativity, and daydreaming*, 386–388. Available online at: http://cdmc.georgetown.edu/wp-content/uploads/2015/03/10-Calvert-HOCPADS-7e-V4-c10_FINAL_2015.pdf (accessed January 12, 2023).

[B11] Calvert S. L. Valkenburg P. M. (2013). “The influence of television, video games, and the internet on children’s creativity,” in *The Oxford handbook of the development of imagination*, ed. Taylor M. (Oxford: Oxford University Press), 438–450. 10.1093/oxfordhb/9780195395761.013.0028

[B12] Cassell J. Ryokai K. (2001). Making space for voice: Technologies to support children’s fantasy and storytelling. *Pers. Ubiquitous Comput*. 5, 169–190. 10.1007/PL00000018

[B13] Chen P. Z. Chang T. C. Wu C. L. (2020). Effects of gamified classroom management on the divergent thinking and creative tendency of elementary students. *Think. Skills Creat*. 36:100664. 10.1016/j.tsc.2020.100664

[B14] Dyachenko O. M. (1996). *The development of the imagination of a preschooler.* Moscow: Mezhdunarodnyi Obrazovatel’nyi i Psikhologicheskii Kolledzh.

[B15] Filippetti V. A. Krumm G. (2020). A hierarchical model of cognitive flexibility in children: Extending the relationship between flexibility, creativity and academic achievement. *Child Neuropsychol.* 26 770–800. 10.1080/09297049.2019.1711034 31913075

[B16] Fleer M. (2022). How conceptual playworlds create different conditions for children’s development across cultural age periods – a programmatic study overview. *New Ideas Child Educ. Psychol.* 2 3–29.

[B17] Gajda A. Karwowski M. Beghetto R. A. (2016). Creativity and academic achievement: A meta-analysis. *J. Educ. Psychol*. 109, 269–299. 10.1037/edu0000133

[B18] Götz M. (2015). “Media, imagination and fantasy,” in *The Routledge international handbook of children, adolescents and media*, ed. Lemish D. (New York, NY: Routledge/Taylor & Francis Group), 186–192.

[B19] Greenfield P. M. (2009). Technology and informal education: What is taught, what is learned. *Science* 323 69–71. 10.1126/science.1167190 19119220

[B20] Greenfield P. M. Farrar D. Beagles-Roos J. (1986). Is the medium the message? An experimental comparison of the effects of radio and television on imagination. *J. Appl. Dev. Psychol.* 7 201–218. 10.1016/0193-3973(86)90029-8

[B21] Hamlen K. R. (2013). Trends in children’s video game play: Practical but not creative thinking. *J. Educ. Comp. Res.* 49 277–291. 10.2190/EC.49.3.a 22612255

[B22] Hutton E. Sundar S. S. (2010). Can video games enhance creativity? Effects of emotion generated by dance dance revolution. *Creat. Res. J.* 22, 294–303. 10.1080/10400419.2010.503540

[B23] Jackson L. A. Witt E. A. Games A. I. Fitzgerald H. E. von Eye A. Zhao Y. (2011). Information technology use and creativity: Findings from the children and technology project. *Comp. Hum. Behav.* 28 370–376. 10.1016/j.chb.2011.10.006

[B24] Kalabina I. A. Progackaya T. K. (2021). Defining digital competence for older preschool children. *Psychol. Russia State Art* 14 169–185. 10.11621/pir.2021.0411 36733823 PMC9888038

[B25] Kannetis T. Potamianos A. Yannakakis G. N. (2009). “Fantasy, curiosity and challenge as adaptation indicators in multimodal dialogue systems for preschoolers,” in *Proceedings of the 2nd Workshop on Child, Computer and Interaction Association for Computing Machinery*, Cambridge, MA. 10.1145/1640377.1640378

[B26] Khokhlova N. I. Muller O. U. Savostina L. V. (2022). Mediation of productive activity as a condition for overcoming computer addiction. *Russ. Psychol. J.* 19 150–160. 10.21702/rpj.2022.2.11

[B27] Krumm G. Arán Filippetti V. Gutierrez M. (2018). The contribution of executive functions to creativity in children: What is the role of crystallized and fluid intelligence? *Think. Skills Creativ.* 29 185–195. 10.1016/j.tsc.2018.07.006

[B28] Linebarger D. L. Barr R. Lapierre M. A. Piotrowski J. T. (2014). Associations between parenting, media use, cumulative risk, and children’s executive functioning. *J. Dev. Behav. Pediatr.* 35 367–377. 10.1097/DBP.0000000000000069 25007059

[B29] Lubart T. I. (1999). “The concept of creativity: Prospects and paradigms,” in *Handbook of creativity*, ed. Sternberg R. J. (London: Cambridge University Press).

[B30] Lucchiari C. Sala P. M. Vanutelli M. E. (2019). The effects of a cognitive pathway to promote class creative thinking. An experimental study on Italian primary school students. *Think. Skills Creat*. 31, 156–166. 10.1016/j.tsc.2018.12.002

[B31] McNeill J. Howard S. J. Vella S. A. Cliff D. P. (2019). Longitudinal associations of electronic application use and media program viewing with cognitive and psychosocial development in preschoolers. *Acad. Pediatr.* 19 520–528. 10.1016/j.acap.2019.02.010 30853576

[B32] Ott M. Pozzi F. (2012). Digital games as creativity enablers for children. *Behav. Inf. Technol*. 31, 1011–1019. 10.1080/0144929X.2010.526148

[B33] Palaigeorgiou G. Katerina K. (2017). “Parental mediation of tablet educational use at home and at school: Facilitators or preventers?,” in *Proceedings of the International Conference on Interactive Mobile Communication, Technologies and Learning (Thessaloniki, Greece, 3 November—1 December, 2017)*, (Cham: Springer), 924–935. 10.1007/978-3-319-75175-7_90

[B34] Papadakis S. (2020). Tools for evaluating educational apps for young children: A systematic review of the Literature. *Interact. Technol. Smart Educ.* 18 18–49. 10.1108/ITSE-08-2020-0127

[B35] Rahimi S. Shute V. J. (2021). First inspire, then instruct to improve students’ creativity. *Comput. Educ*. 174:104312. 10.1016/j.compedu.2021.104312 36569795 PMC9758891

[B36] Runco M. A. Jaeger G. J. (2012). The standard definition of creativity. *Creativ. Res. J.* 24 92–96. 10.1080/10400419.2012.650092

[B37] Singer D. G. Singer J. L. (2005). *Imagination and play in the electronic age*. Cambridge, MA: Harvard University Press.

[B38] Soldatova G. U. Vishneva A. E. (2019). Features of the development of the cognitive sphere in children with different online activities: Is there a golden mean? *Counsel. Psychol. Psychother.* 27 97–118. 10.17759/cpp.2019270307

[B39] Sternberg R. J. Lubart T. I. (1999). “The concept of creativity: Prospects and paradigms,” in *Handbook of creativity*, ed. Sternberg R. J. (Cambridge: Cambridge University Press). 10.1017/CBO9780511807916.003

[B40] Strouse G. A. O’Doherty K. Troseth G. L. (2013). Effective co-viewing: Preschoolers’ learning from a video after a dialogic questioning intervention. *Dev. Psychol.* 49 2368–2381. 10.1037/a0032463 23544859

[B41] Torrance E. P. (1962). *Guiding creative talent.* Hoboken, NJ: Prentice Hall. 10.1037/13134-000

[B42] Tvardovskaya A. A. Gabdulkhakov V. F. Novik N. N. (2022). Bilingualism and executive functions in preschoolers: A review of the research progress. *Uchenye Zapiski Kazanskogo Univ. Seriya Gumanitarnye Nauki* 164 87–100. 10.26907/2541-7738.2022.1-2.87-100

[B43] Valkenburg P. M. van der Voort T. (1995). The influence of television on children’s daydreaming styles. *Commun. Res*. 22, 267–287.

[B44] Veraksa A. N. Almazova O. V. Bukhalenkova D. A. Gavrilova M. N. (2020). Possibilities of using game roles for training regulatory functions in preschoolers. *Cult. Hist. Psychol.* 1 111–121. 10.17759/chp.2020160111

[B45] Veraksa A. N. Kornienko D. S. Chichinina E. A. Bukhalenkova D. A. Chursina A. V. (2021). Correlations between preschoolers’ screen time with gender, age and socio-economic background of the families. *Art Sci. Televis.* 17 179–209. 10.30628/1994-9529-17.3-179-209

[B46] Veraksa N. E. Veraksa A. N. Gavrilova M. N. Bukhalenkova D. A. Oshchepkova E. S. Chursina A. V. (2021). Short-and long-term effects of passive and active screen time on young children’s phonological memory. *Front. Educ.* 6:600687. 10.3389/feduc.2021.600687

[B47] Veraksa N. Bukhalenkova D. Chichinina E. Veraksa A. Saljo R. (2022). “Use of digital devices and child development: Digital tools or digital environment? a cultural–historical perspective,” in *Child development in Russia: Perspectives from an international longitudinal study*, ed. Veraksa A. (Cham: Springer International Publishing), 159–180. 10.1007/978-3-031-05524-9_8

[B48] Vygotsky L. S. (1984). “Child psychology,” in *Collected works in 6 t*, Vol. 4 ed. Zaporozhets A. V. (Moscow: Pedagogy).

[B49] Williams R. Runco M. A. Berlow E. (2016). Mapping the themes, impact, and cohesion of creativity research over the last 25 years. *Creativ. Res. J.* 28 385–394. 10.1080/10400419.2016.1230358

[B50] Xiong Z. Liu Q. Huang X. (2022). The influence of digital educational games on preschool children’s creative thinking. *Comp. Educ.* 189:104578. 10.1016/j.compedu.2022.104578

[B51] Yildiz C. Yildiz T. G. (2021). Exploring the relationship between creative thinking and scientific process skills of preschool children. *Think. Skills Creativ.* 39:100795. 10.1016/j.tsc.2021.100795

[B52] Yudina E. G. (2022). Pretend play as the territory of freedom. *Nat. Psychol. J.* 3 13–25. 10.11621/npj.2022.0303

